# Evaluation of coagulation with TEG in patients diagnosed COVID-19

**DOI:** 10.3906/sag-2106-379

**Published:** 2021-10-30

**Authors:** Hülya VATANSEV, Mehmet Ali KARASELEK, Resül YILMAZ, Serkan KÜÇÇÜKTÜRK, Ahmet TOPAL, Şebnem YOSUNKAYA, Adem KÜÇÜK, Celalettin VATANSEV

**Affiliations:** 1 Department of Chest Diseases, Meram Medicine Faculty, Necmettin Erbakan University, Konya, Turkey; 2 Department of Internal Medicine, Meram Medicine Faculty, Necmettin Erbakan University, Konya, Turkey; 3 Department of Anesthesiology and Reanimation, Meram Medicine Faculty, Necmettin Erbakan University, Konya, Turkey; 4 Department of Medical Biology, Medicine Faculty, Karamanoğlu Mehmetbey Univesity, Karaman, Turkey; 5 Department of General Surgery, Meram Medicine Faculty, Necmettin Erbakan University, Konya, Turkey

**Keywords:** Thromboelastography, COVID-19, D-Dimer, coagulopathy, thrombosis

## Abstract

**Background and aim:**

A high D-dimer level may indicate the risk of coagulopathy and mortality in COVID-19 patients. Thromboelastography (TEG) is a test that evaluates clot formation and fibrinolysis in real-time, unlike routine coagulation tests. The study aimed to investigate the coagulation process with TEG in patients diagnosed with COVID-19.

**Materials and Methods:**

The study was performed at our university hospital, chest diseases outpatient clinic as a cross-section study. A total of 51 patients with 23 high D-dimer levels group (HDG) and 28 low D-dimers group (LDG) were included in the study. TEG analysis was performed at the pretreatment evaluation in these two groups.

**Results:**

D-dimer and fibrinogen levels of the HDG were higher than those of the LDG (550 vs. 90 ng/mL, p < 0.001; 521 vs. 269 mg/dL, p < 0.001, respectively). In TEG analysis, HDG’s R and K values were lower than LDG, and HDG’s Angle, MA, and CI values were higher than LDG (p = 0.037; p < 0.001; p < 0.001; p < 0.001; p < 0.001, respectively). ROC curve analysis suggested that the optimum TEG parameters cut-off points for thrombosis risk were as below: for K was ≤2.1 min, for R was ≤6.1 min, for Angle was >62°, MA was 60.4 mm.

**Conclusion:**

Our study showed that the risk of thrombosis might increase in COVID-19 patients who are not hospitalized in the intensive care unit. Thrombosis risk should be investigated with TEG analysis and laboratory tests in every patient diagnosed with COVID-19, and treatment should be started for risky patients.

## 1. Introduction

The world experienced two global pandemics, severe acute respiratory syndrome (SARS) in 2002–2003 and Middle East Respiratory Syndrome (MERS) in 2011. The cause of both outbreaks has been identified as a beta coronavirus (CoV) of zoonotic origin. At the end of 2019, “Corona Virus 2019 Disease (COVID-19)”, caused by a new type of beta coronavirus called SARS-CoV-2, emerged as a new pandemic in the city of Wuhan, China [1].

The SARS-CoV-2 virus causes damage to many organs, especially the lung, and venous and arterial thrombotic events are pervasive in patients with COVID-19 [2,3]. These thrombotic events may be the result of hypercoagulation caused by the inflammatory reaction caused by SAR-CoV-2. A recent study reported that approximately 20% of patients with COVID-19 developed venous thromboembolism (VTE), with a cumulative incidence of VTE reaching 49% during their hospitalization [4]. Studies have also consistently reported a significant increase in D-dimer value. High D-dimer levels indicate that coagulopathy plays a vital role in the prognosis of the disease and has a remarkable impact on mortality [5]. Evaluation of the coagulation status may be crucial for the disease prognosis.

Thromboelastography (TEG) is a test that evaluates clot formation and fibrinolysis in real-time, unlike conventional coagulation tests [6,7]. Because of this, TEG can help detect thromboembolic events in the patient. Recent studies have reported different hypercoagulation profiles in TEG analyzes of patients with COVID-19 [2,3,8,9]. Chronic and infectious diseases and their course may show genetic and ethnic differences between societies. Therefore, the current study aimed to evaluate the coagulation process with TEG in patients diagnosed with COVID-19 by real-time polymerase chain reaction (RT-PCR) in the Turkish population. As secondary implications, we aimed to identify risk factors for embolic events in COVID-19 and to determine the character of coagulation with data obtained from TEG analysis.

## 2. Materials and Methods

### 2.1. Patients

**Figure 1:**
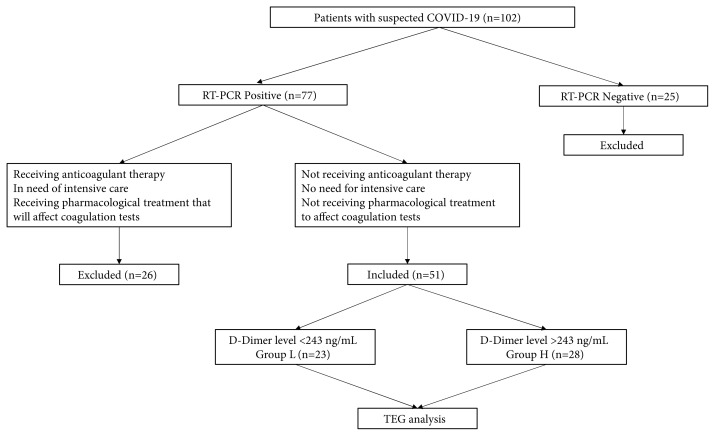
A flow diagram of the study design.

This cross-section study was conducted by the Declaration of Helsinki, with the approval of the local ethics committee (2020/2779), between October 2020 and December 2020 in Necmettin Erbakan University, Meram Medical Faculty Hospital Chest Diseases outpatient clinic. Throat swab samples were evaluated using the RT-PCR method, and SARS-CoV-2 positivity was detected. The study included 51 patients with positive RT-PCR tests and no previous diagnosis of COVID-19 and no treatment for COVID-19. The exclusion criteria were as follows: 1) need for intensive care unit support in the last ten days; 2) taking any anticoagulant therapy; 3) using any pharmacological treatment that affects coagulation tests. Patients excluded from the study population during the study period are presented in Figure 1 as a flowchart.

### 2.2. Study design

Demographic data (age, gender, smoking, alcohol, and chronic diseases) obtained during the application process, laboratory results (white blood count cell [WBC], lymphocyte, platelets [PLT], D-dimer, fibrinogen, ferritin, C-reactive protein [CRP], lactate dehydrogenase [LDH]), and hospital duration and mortality ratio were recorded in the case study file. The lung involvement of the patients was evaluated by computed tomography (CT) (Siemens Samatom Drive 256, Germany). Lung CT images of patients with COVID-19 were classified as negative, atypical, indistinct, and typical [10]. In addition to laboratory tests, TEG analysis was performed by taking 3 ml of a peripheral blood sample from the brachial vein into a citrated blood sampling tube. According to the D-dimer laboratory results, the cases were divided into two groups as high and low. Patients with D-Dimer level <243 ng/mL were classified as low D-Dimer group (LDG) and patients with >243 ng/mL level as high D-Dimer group (HDG).

### 2.3. Thromboelastography analysis

TEG analysis was performed with TEG 5000, version 4.2 (Haemoscope Corporation, Niles, IL, USA). 1 ml of whole blood was taken from the citrate tube and transferred to the kaolin tube. The blood was gently mixed for complete contact with kaolin. Kaolin and whole blood were taken from the mixture with a 340 μl automatic straw and placed in the test tube. For antagonize citrate, 20 μl of calcium chloride was added to the test tube with an automatic straw, and the test was analyzed, and its results were interpreted. As a result of the analysis, reaction time (R, minute), clot formation time (K, minute), degree (Angle), maximum amplitude (MA, mm), and coagulation index (Cl) values were obtained. R is the clot formation time; K is the time taken for the amplitude of 2 mm to increase to 20 mm; The alpha angle represents the clot formation rate, and the MA represents the clot strength.

### 2.4. Statistical analysis

Statistical tests were performed using statistical software Jamovi version 1.2.27 (The Jamovi project 2021, Sydney, Australia). The distribution of the data was analyzed using the Kolmogorov Smirnov test. An independent t-test was used for the intergroup comparison of variables with normal distribution. The data of these results were expressed as mean ± standard error. For variables that do not show normal distribution, the Mann-Whitney U test was applied for intergroup comparison. These values are also shown in the tables as median (first quarter-third quarter). The Spearman correlation test was used to determine the correlation between D-Dimer and TEG parameters. The statistical software MedCalc version 18.9.1 (MedCalc software bv, Ostend, Belgium) was used for the receive operating characteristic (ROC) analysis. The area under the curve (AUC) and the 95% confidence interval (CI) were calculated using ROC analysis. Youden’s J statistic determined the optimal cut-off points. A p value <0.05 was considered statistically significant.

## 3. Results

The male/female ratio of 51 patients was 25/26, mean age was 53.1±4.1 / 51.8±3.8 years, respectively (Table 1).

As a result of the comparison of TEG parameters; HDG’s R time and K value were lower than LDG, and HDG’s Angle, MA, and CI values were higher than LDG (respectively; p = 0.037; p <0.001; p <0.001; p <0.001; p <0.001) (Table 2).

D-dimer and fibrinogen levels of the HDG were remarkably higher than those of the LDG (550 ng/mL vs. 90 ng/mL, p < 0.001; 521 mg/dL vs. 269 mg/dL, p < 0.001, respectively). The ferritin, CRP, and LDH values of HDG were significantly higher than those of LDG, while the lymphocyte count of HDG was significantly lower than HDG (all p < 0.05). The hospital length of stay of HDG was higher than that of LDG (8 days vs. one day, p < 0.001). In the follow-up of all patients after the 10th day, no worsening was observed in the LDG. But two patients in the HDG were taken to the intensive care unit, and one of them died of sepsis-related multiorgan failure (Table 3).

As a result of the correlation analysis between D-Dimer and TEG parameters, other TEG parameters were statistically significant except for R (r = –0.185; p = 0.19). K was strongly negative, angle, MA and Cl were strongly positively correlated (respectively; r = –0.72, p < 0.001; r = 0.64, p < 0.001; r = 0.69, p < 0.001; r = 0.65, p < 0.001).

**Figure 2:**
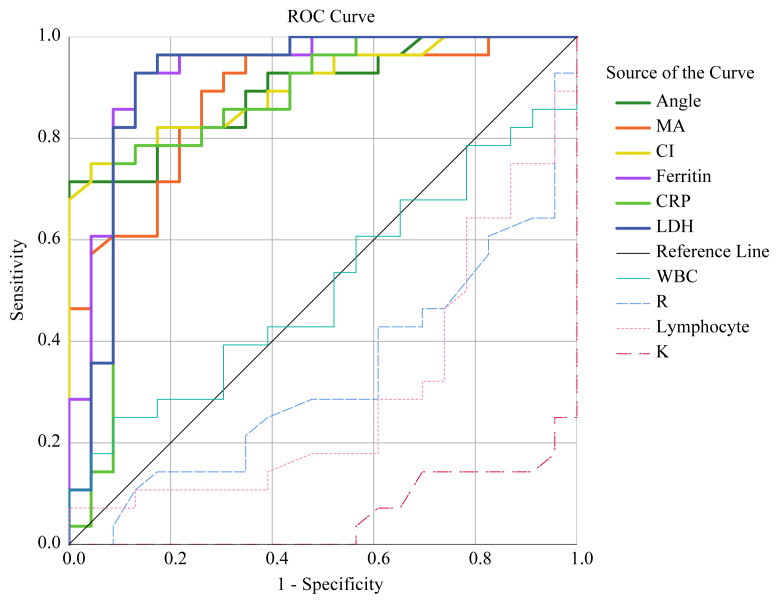
ROC curves by high D-Dimer levels.

ROC curve analysis suggested that the optimum TEG parameters cut-off points for thrombosis risk were as below: for K was ≤2.1 min, for R was ≤6.1 min, for Angle was >62°, MA was 60.4 mm. All AUC, Cl, optimum cut-off values, sensitivity, and specificity values are given in Table 4 and Figure 2.

According to the calculated cut-off values, the patients were divided into two groups as high and low risk for thrombosis. TEG parameters between the two groups were statistically significant (Table 5).

## 4. Discussion

Coagulopathy may occur during patients with COVID-19. The lung is the primary organ affected in this disease, but multiorgan failure may also develop. A significant increase in D-dimer and fibrin/fibrinogen degradation products in COVID-19 is the first sign of the onset of the coagulopathy event. At this stage, there are usually no abnormalities in prothrombin time, partial thromboplastin time, and platelet counts [4]. Therefore, although it is recommended to monitor D-dimer and fibrinogen levels in patients, many centers start anticoagulant treatment as prophylactic [11]. However, VTE may occur even under anticoagulant therapy [5,11–13]. Therefore, evaluation of the coagulation mechanisms of patients with COVID-19 via TEG may help in the early diagnosis and selection of appropriate anticoagulant therapy. TEG analysis can show the presence of hypercoagulation earlier than laboratory tests. Low K and high angle and MA values indicate hypercoagulation and vice versa show hypocoagulation [7]. According to the results of TEG analysis in the current study, Angle and MA values of HDG were higher than LDG, and the K value of HDG was lower than LDG. TEG results of our study were consistent with the presence of hypercoagulation without any symptoms in the patients. Correlation analysis revealed a positive relationship between TEG results and high D-dimer levels in our findings. According to our findings, COVID-19 patients with high D-dimer levels were more prone to coagulation than COVID-19 patients with low D-dimer levels. Recently, TEG analysis in several studies has shown that COVID-19 patients followed in the intensive care unit are prone to hypercoagulation [8, 12–14]. These studies were carried out in patients diagnosed with severe COVID-19 and followed up in the intensive care unit [3,4,7–9]. The design of our research is entirely different from previous studies. In the current study, it has been shown that hypercoagulability may occur in COVID-19 patients who do not require intensive care unit follow-up at the time of diagnosis and during the ten-day follow-up period.

High D-dimer and fibrinogen levels in patients with COVID-19 are closely associated with an increased risk of developing thromboembolism, poor prognosis, and increased mortality [11,15]. Our study showed that patients with high D-Dimer values had a lengthy hospital stay than patients with low D-Dimer values. In the follow-up of HDG patients after ten days, two patients required intensive care support, and one of them died due to sepsis and its complications. Previous studies have reported age, lymphopenia, increased CRP, LDH, and ferritin levels as predictive markers for poor prognosis in patients with COVID-19 [16,17]. We found that HDG had lower lymphocyte counts and higher CRP, LDH, and ferritin levels than LDG. We also found that this change in laboratory findings was associated with hypercoagulopathy. Detection of the hypercoagulopathy problem before thrombosis occurs may be vital in patients with COVID-19. Therefore, TEG analysis can be a crucial diagnostic and follow-up method in these patients. We detected a high risk of thrombosis in patients according to TEG analysis. We obtained predictive optimum cut-off values of TEG parameters for hypercoagulopathy by ROC analysis. Our study may reveal that there is a risk of thrombosis even in patients with COVID-19 who are not followed up in the intensive care unit and that early follow-up may reduce complications and mortality in these patients.

We found that TEG values ​​(low K and R-value and high MA and angle value) and increased laboratory values ​​(ferritin, LDH) were associated with D-dimer level. In addition, we found that TEG values ​​could be a predictive marker for thrombosis risk at high sensitivity and specificity values. Previous studies have demonstrated that VTE can occur even under standard thromboprophylaxis therapy in patients with COVID-19 [4, 7]. Some studies recommend TEG to detect the risk of coagulation in patients with severe COVID-19 and to adjust the dose of anticoagulant drugs for thrombosis prophylaxis and treatment [18]. A metaanalysis showed that heparin treatment might reduce the length of stay hospital and mortality rate in critically ill COVID-19 patients in intensive care units [19]. Our study revealed that heparin or other anticoagulants could be required for thrombosis prophylaxis in patients with COVID-19 who do not need intensive care.

Standard heparin and low molecular weight heparin are recommended for anticoagulant selection. Heparin affects the onset of coagulation [20,21]. As a result of literature information and TEG analysis results, the shorter R and K times and the large angle indicate that heparin is the right choice in HDG. However, the high MA in HDG cases is associated with high PLT activation and an intense clot and indicates the prothrombotic state. Patients with COVID-19 who do not receive intensive care unit support with high D-Dimer value, and TEG analysis results may also need to receive heparin therapy. Our study is a pilot study. There is a need for detailed randomized large population studies on this subject.

## 5. Conclusion

Heparin treatment reduces the length of hospital stay and mortality rate in patients with COVID-19 in the intensive care unit. TEG analysis is beneficial in the early detection of thrombosis risk. Our study showed that the risk of thrombosis might increase in COVID-19 patients who are not hospitalized in the intensive care unit. Thrombosis risk should be investigated with TEG analysis and laboratory tests in every patient diagnosed with COVID-19, and treatment should be started for risky patients. However, our study is a pilot study, and detailed further studies are needed.

## Figures and Tables

**Table 1:** Age-sex distribution of the patients.

	HDG Mean+SEM (n)	LDG Mean+SEM (n)	p value
**Age (Years)**	62.5 ± 2.6 (28)	40.2 ± 4.0 (23)	<0.001
**Female (Years)**	62.9 ± 3.2 (14)	38.9 ± 5.4 (12)	0.001
**Male (Years)**	62.1 ± 4.1 (14)	41.6 ± 6.3 (11)	0.009

(HDG, high D-dimer group; LDG, low D-Dimer group).

**Table 2:** TEG results of patients diagnosed with COVID-19

Parameters	HDG (n = 28)	LDG (n = 23)	
	Median	Median	p value
**R (min)**	5.0 (4.0–6.8)	6.6 (5–7.5)	0.037
**K (min)**	1.7 (1.4–2.0)	4 (2.7–5.1)	<0.001
**Angle (**°**)**	65.4 (60.1–69.7)	48.5 (34.2–55.9)	<0.001
**MA (mm)**	67.8 (62.3–72.1)	54.4 (49.3–60.9)	<0.001
**CI**	1.4 (0.3–2.5)	-2.5 (-5.3–0.9)	<0.001

(TEG, Thromboelastography; HDG, high D-dimer group; LDG, low D-Dimer group; R, reaction time; K, clot formation time; Angle, degree; MA, maximum amplitude; Cl, coagulation index; min, minute; mm, millimeter). Results expressed as Median (1. Quarter – 3. Quarter)

**Table 3:** Laboratory results of patients diagnosed with COVID-19.

Parameters	HDG (n = 28)	LDG (n = 23)	p value
	Median	Median	
**D-dimer (ng/mL)**	550.0 (427.0–903.0)	90 (52–155)	**<0.001**
**Fibrinogen (mg/dL)**	521.0 (431.5–618.3)	269 (221–382)	**<0.001**
**PLT (10** **^3^** ** /uL)**	252.5 (159.5–322.8)	244 (184–295)	0.895
**Total Hospitalization (Day)**	8 (5.25–10)	1 (1–4)	**<0.001**
**WBC (10** **^3^** **/uL)**	6.59 (5.3–9.7)	6.82 (5.5–8.2)	0.890
**Lymphocyte (10** **^3^** **/uL)**	0.92 (0.65–1.44)	1.73 (0.9–2.4)	**0.019**
**Ferritin (ug/L)**	369.6 (180.5–842.1)	40.2 (11.7–95.9)	**<0.001**
**CRP (mg/L)**	72.39 (32.7–108.7)	5.01 (1.92–21.1)	**<0.001**
**LDH (U/L)**	328.5 (277.3–398.3)	195 (164–218)	**<0.001**

(HDG, high D-dimer group; LDG, low D-Dimer group; PLT, platelets; WBC, white blood cell counts; CRP, C-reactive protein; LDH, lactate dehydrogenase). Results expressed as Median (1. Quarter – 3. Quarter)

**Table 4:** ROC analysis results for thrombosis risk.

Parameters	AUC	95% CI	Sensitivity	Specificity	Cut-off	p value
**K (min)**	0.94	0.84–0.99	82.1	95.7	≤2.1	<0.001
**Ferritin (ug/L)**	0.93	0.82–0.98	93	87	>125.8	<0.001
**LDH (U/L)**	0.91	0.80–0.97	93	87	>241	<0.001
**Angle (**°**)**	0.90	0.78–0.96	71.4	100	>62	<0.001
**CI**	0.90	0.79–0.97	75	96	>0.4	<0.001
**MA (mm)**	0.88	0.76–0.95	89	74	>60.4	<0.001
**CRP (mg/L)**	0.85	0.72–0.93	75	91.3	>34.8	<0.001
**Lymphocyte (10** **^3^** **/uL)**	0.71	0.56–0.83	82.1	61	≤1.5	<0.006
**R (min)**	0.67	0.53–0.80	71.4	61	≤6.1	0.024
**WBC (10** **^3^** **/uL)**	0.51	0.36–0.65	25	91.3	>8.8	0.960

(AUC, area under the curve; Cl, confidence intervals; K, clot formation time; LDH, lactate dehydrogenase; Cl, coagulation index; MA, maximum amplitude; CRP, C-reactive protein; R, reaction time; WBC, white blood cell counts).

**Table 5:** Comparison of patients separated according to cut-off values in terms of TEG parameters.

Parameters	Cut-off	n	Median	p value
**R (min)**	>6.1	23	7.3 (6.7–8.3)	**<0.001**
	≤6.1	28	4.8 (3.9–5.1)	
**K (min)**	>2.1	29	3.3 (2.6–4.4)	**<0.001**
	≤2.1	22	1.6 (1.3–1.8)	
**Angle (**°**)**	>62	20	49.7 (41.7–57.5)	**<0.001**
	≤62	31	67.0 (65.0–70.2)	
**MA (mm)**	>60.4	31	67.3 (63.0–71.5)	**<0.001**
	≤60.4	20	52.4 (48.2–58.3)	
**CI**	>0.4	23	1.8 (0.9–2.7)	**<0.001**
	≤0.4	28	–2.5 (from –5.2 to –1.3)	

(TEG, Thromboelastography; R, reaction time; K, clot formation time; Angle, degree; MA, maximum amplitude; Cl, coagulation index; min, minute; mm, millimeter). Results expressed as Median (1. Quarter - 3. Quarter)
